# Relationship between Aortic Compliance and Impact of Cerebral Blood Flow Fluctuation to Dynamic Orthostatic Challenge in Endurance Athletes

**DOI:** 10.3389/fphys.2018.00025

**Published:** 2018-01-25

**Authors:** Tsubasa Tomoto, Tomoko Imai, Shigehiko Ogoh, Seiji Maeda, Jun Sugawara

**Affiliations:** ^1^Human Informatics Research Institute, National Institute of Advanced Industrial Science and Technology, Ibaraki, Japan; ^2^Center for General Education, Aichi Institute of Technology, Toyota, Japan; ^3^Department of Biomedical Engineering, Toyo University, Kawagoe, Japan; ^4^Faculty of Health and Sport Sciences, University of Tsukuba, Tsukuba, Japan

**Keywords:** cerebral hemodynamics, aortic compliance, endurance training, pulsatile blood flow, lower body negative pressure stimulation

## Abstract

Aorta effectively buffers cardiac pulsatile fluctuation generated from the left ventricular (LV) which could be a mechanical force to high blood flow and low-resistance end-organs such as the brain. A dynamic orthostatic challenge may evoke substantial cardiac pulsatile fluctuation via the transient increases in venous return and stroke volume (SV). Particularly, this response may be greater in endurance-trained athletes (ET) who exhibit LV eccentric remodeling. The aim of this study was to determine the contribution of aortic compliance to the response of cerebral blood flow fluctuation to dynamic orthostatic challenge in ET and age-matched sedentary (SED) young healthy men. ET (*n* = 10) and SED (*n* = 10) underwent lower body negative pressure (LBNP) (−30 mmHg for 4 min) stimulation and release the pressure that initiates a rapid regain of limited venous return and consequent increase in SV. The recovery responses of central and middle cerebral arterial (MCA) hemodynamics from the release of LBNP (~15 s) were evaluated. SV (via Modeflow method) and pulsatile and systolic MCA (via transcranial Doppler) normalized by mean MCA velocity (MCAv) significantly increased after the cessation of LBNP in both groups. ET exhibited the higher ratio of SV to aortic pulse pressure (SV/_Ao_PP), an index of aortic compliance, at the baseline compared with SED (*P* < 0.01). Following the LBNP release, SV was significantly increased in SED by 14 ± 7% (mean ± SD) and more in ET by 30 ± 15%; nevertheless, normalized pulsatile, systolic, and diastolic MCAv remained constant in both groups. These results might be attributed to the concomitant with the increase in aortic compliance assessed by SV/_Ao_PP. Importantly, the increase in SV/_Ao_PP following the LBNP release was greater in ET than in SED (*P* < 0.01), and significantly correlated with the baseline SV/_Ao_PP (*r* = 0.636, *P* < 0.01). These results suggest that the aortic compliance in the endurance athletes is able to accommodate the additional SV and buffer the potential increase in pulsatility at end-organs such as the brain.

## Introduction

Exaggerated hemodynamic fluctuation would be a profound mechanical force to high blood flow and low-resistance end-organs (e.g., the brain and kidneys) (O'rourke and Safar, 2005; Mitchell, 2008). Normally, central elastic arteries (e.g., aorta and carotid artery) effectively buffer cardiac pulsatile fluctuation generated from the left ventricular (LV) (Nichols and McDonald, 2011). Conversely, the impaired buffering ability of central elastic arteries leads to chronic exposure to mechanical stress which could be a strong risk factor for cerebrovascular disease (e.g., white-matter damage and lacuna stroke) (Bateman, 2004; London and Pannier, 2010; Tuttolomondo et al., 2010; Tarumi et al., 2014; Wahlin et al., 2014). In addition, smaller cardiac ejection (e.g., SV) is likely to be associated with smaller cerebrovascular hemodynamic fluctuation and vice versa (Sugawara et al., 2017).

It is well-known that chronic endurance training induces the eccentric remodeling (e.g., increased chamber size) and superior compliance characteristics of the LV (Levine et al., 1991; Pluim et al., 2000; Scharhag et al., 2002; Caselli et al., 2011; Tomoto et al., 2015). Previously, these cardiac adaptations with superior aortic compliance in endurance athletes were reported (Dupont et al., 2017). As stroke volume (SV) increases in response to an increase in the end-diastolic volume which synchronized with the venous return (Guyton and Hall, 2011), such individuals exhibit a greater increase in SV after volume loading. Postural alteration evokes drastic gravitational hemodynamic changes such as greater pulsatile fluctuations of blood flow and blood pressure. In this context, postural alteration-related volume loading (e.g., increased venous return) may cause the substantial change in SV in endurance-trained athletes. However, it is unknown the impact of rapid postural change on central and cerebral hemodynamics in endurance-trained individuals.

The aim of this study was to test the hypothesis that, in endurance-trained athletes who have greater arterial compliance of the proximal aorta, postural alteration-induced substantial increase in SV does not cause the augmented pulsatile fluctuation of cerebral blood flow (CBF). To test our hypothesis, we applied lower body negative pressure (LBNP) release technique as previously used to elicit an immediate increase in central blood volume and blood pressure (Zhang et al., 1998; Ogoh et al., 2015).

## Methods

### Subjects

Twenty young healthy men voluntarily participated in this investigation. Each subject received a verbal and written explanation of the associated objectives, techniques of measurement and risks. In accordance with the Declaration of Helsinki, each subject provided written informed consent for participation and all protocols were approved by the Ethics Committee of the Human Research Institutional Committee of the National Institute of Advanced Industrial Science and Technology (No. 2013-434). Group of endurance-trained (ET, *n* = 10) and age-matched sedentary (SED, *n* = 10) subject were stratified according to their recent history of exercise training. ET had been participating in regular aerobic endurance training for 12 ± 4 h/week (mainly endurance exercise training) and engaging long distance running and/or swimming for 10 ± 2 years; while, untrained subjects did not regularly partake in regular aerobic and resistance exercise (1 ± 1 h/week). All subjects were healthy, normotensive (<140/90 mmHg), nonobese (body mass index, BMI < 25 kg/m^2^), nonsmokers, free of medication, overt chronic heart and lung diseases, head trauma as assessed by medical history. None of the subjects were taking cardiovascular-acting medication. Prior to the experimental sessions, each subject was familiarized with the equipment and the experimental protocol. Subjects were requested to abstain from food intake for 3 h, caffeinated beverages for at least 12 h, and strenuous physical activity and alcohol intake for at least 24 h before the testing. The subjects were allowed to drink water to keep well hydration.

### Experimental protocol

Experimental measures including body composition, cardiac echocardiography at rest, and the response of cerebral blood flow velocity (CBFV) and arterial blood pressure at rest, during 30 mmHg negative pressure (LBNP −30 mmHg) and after release up to 15 s. All measurements were conducted in an environmentally controlled laboratory with a quiet and air-conditioned room (24–25°C).

After arrival at the laboratory, the subjects underwent body composition assessment (e.g., weight and height), and supine rest at least 20 min. After cardiac data acquirement and instrumentation for LBNP testing, each participant was placed in a supine position inside the LBNP testing chamber. Once inside, they straddled a wood seat with their feet clear of the base of the chamber and sealed at the level the iliac crest. Following 6 min rest at ambient barometric pressure, negative pressure was gradually induced using a commercially available vacuum and quantified with pressure transducer was invoked. The LBNP test was terminated when the participant completed 4 min at LBNP −30 mmHg. During this experimental protocol, negative pressure was gradually increased and carefully monitored the signs of impending presyncope include dizziness, nausea, profuse sweating, or a rapid change in blood pressure defined as either a decrease in systolic blood pressure by 25 mmHg or a decrease in diastolic blood pressure by 15 mmHg within 1 min. When these signs were reported or observed, the experimental was stopped. These experimental procedures have been demonstrated in our previous research (Sugawara et al., 2017).

#### Anthropometric parameter

For the anthropometric parameters, BMI measurement was calculated by the formula weight in weight/height. The body surface area (BSA) was obtained using the formula: √(height×weight)/3,600 (Mosteller, 1987).

#### Cardiovascular measurements

Echocardiographic examinations were performed in the left lateral decubitus position using a CX50 xMATRIX (Philips Ultrasound., Bothell, WA) equipped with a multifrequency probe (2.5-MHz transducer). The end-diastolic interventricular septum (IVST) and LV posterior wall thicknesses (PWT) and LV end-diastolic dimension (LVEDd) and end-systolic dimensions (LVESd) were measured on M-mode images in the long parasternal view. LV ejection fraction (EF) and LV fractional shortening (FS) were calculated by Teichholz's method (Teichholz et al., 1976). Doppler velocity time integral (VTI) was obtained from LV ejection velocity wave form recorded via an apical three-chamber view. SV was computed from multiplying the VTI by cross sectional area of LV outflow tract (LVOT) via parasternal long axis view, as previously reported (Lewis et al., 1984). LVOT diameter was measured from the distance between the base of aortic valve right after the valve was fully open. Left ventricular mass (LVM) was calculated following equation: LVM (g) = 0.8 × [1.04 × (IVST + LVEDd + PWT)^3^ − (LVEDd)^3^]+ 0.6. (Devereux et al., 1986). LVM index (LVMi) was normalized LVM by BSA. Electrocardiographic (ECG) data were recorded using a three-lead system for calculation of heart rate (ML 132 Bio Amp, ADInstruments Inc., Colorado Springs, CO). Radial arterial pressure waveforms were continuously recorded at the left wrist by a validated applanation tonometry-based automated radial blood pressure waveform measurement device (Jentow, Nihon Colin Co, Komaki, Japan), and those were calibrated with oscillometry-derived brachial mean and diastolic blood pressures. Beat-by-beat SV was derived from radial arterial pressure waveforms via the Modelflow method (BeatScope 1.1a, Finapres Medical System BV, Amsterdam, the Netherlands), and which was calibrated by the reference value of SV by Doppler echocardiography technique so that the baseline Modelflow-derived SV was made equal to the reference value in each subject. Cardiac output was calculated by multiplying SV by heart rate. Systemic vascular conductance was computed by dividing cardiac output by mean arterial pressure. Beat-to-beat aortic (Ao) blood pressure waveforms were computed from radial arterial pressure waveforms by a validated generalized transfer function technique (SCOR-Mx, SphygmoCor, AtCor Medical, Sydney, Australia), as we previously reported (Sugawara et al., 2017). To determine the aortic compliance, SV/_Ao_PP was calculated where _Ao_PP is pulse pressure at proximal aorta.

#### Cerebral vascular hemodynamics

CBFV was continuously measured at least 6 min at middle cerebral artery (MCA) over the temporal window ipsilateral using 2-MHz transcranial Doppler (TCD) probe (EZ Dop; DWL, Sipplingen, Germany.) The sampling depth was set from 42 to 55 mm, and the angle of the Doppler probe and the sampling depth were adjusted to optimize the signal quality for each subject according to standard procedures (Alexandrov et al., 2012). During data collection, subjects were instructed to breathe normally. The partial pressure of end-tidal carbon dioxide (P_ET_CO_2_) was monitored by a metabolic cart equipped with a respiratory gas analyzing system (AE280S; Minato Medical Science, Tokyo, Japan). Doppler signal was stored at 200 Hz with an acquisition system (PowerLab 8/30, ADInstruments, Colorado Springs, CO, USA) interfaced with a personal computer equipped with data acquisition software (LabChart 7.1, ADInstruments, Colorado Springs, CO, USA). Beat-to-beat time average velocity (e.g., mean velocity), systolic MCA velocity (MCAv), diastolic MCAv, and pulsatile (=systolic − diastolic) MCAv were obtained from 6 min for baseline, 4 min for LBNP testing, and 15 s for releasing LBNP stimulation by offline analysis, and the averaged values of each parameter were reported. Because normalized values of MCA systolic, diastolic, and pulsatile for mean (i.e., time-averaged) velocity (%) (%systolic MCAv, %diastolic MCAv, and %pulsatile MCAv, respectively) have reported with strong correlation with carotid compliance (Tomoto et al., 2015) and brain structures (Tarumi et al., 2014) in previous research, we also obtained these MCAv% parameters. The pulsatile indices of MCA were calculated from the following equations:

$$\begin{array}{l} \text{ Systolic MCAv}\%=(\text{Absolute systolic MCA velocity}\\ \;/\text{time averaged MCA velocity})\;\times 100\\ \text{Diastolic MCAv}\%=(\text{Absolute diastolic MCA velocity}\\ \;/\text{time averaged MCA velocity})\;\times 100\\ \text{ Pulsatile MCAv}\%=(\text{Absolute pulsatile MCA velocity}\\ \;/\text{time averaged MCA velocity})\;\times 100 \end{array}$$

Cerebrovascular resistance index was calculated as a ratio of mean radial arterial pressure to mean MCA velocity (MCAv).

### Statistics

Students' independent *t*-test by groups was performed to determine the impact of continuous endurance training habits on variables of interest and the magnitude difference of recovery responses in central and cerebral hemodynamics from the release of LBNP. Simple correlation analysis was performed to identify the effect of aortic compliance at baseline to the response to the LBNP release. Statistical comparisons of variables were made utilizing a repeated-measures two-way analysis of variance (ANOVA) with a 3 × 2 design (LBNP × group). A Student-Newman-Keuls test was employed *post-hoc* when interactions were significant. Statistical significance was set at *P* < 0.05 and results are present as mean ± SD.

## Results

### General observations

Subjects' anthropometric parameters, systemic hemodynamics, and LV parameters are shown in Table 1. Anthropometric parameters did not significantly differ between the SED and ET. In systemic hemodynamics, arterial pressures did not differ between groups. Whereas heart rate was significantly lower and SV was significantly larger in ET, thereby, cardiac output did not differ between groups. In LV characteristics, LVEDd, LVESd, LVM, LVMi, PWT, and IVST were significantly greater in ET than SED, as expected. Whereas, LV fractional shortening and ejection fraction showed no differences between each group. In cerebrovascular parameters, mean and diastolic MCAv did not differ between the groups; whereas, the systolic and pulsatile MCAv in ET were significantly lower than SED (Table 2) with higher SV/_Ao_PP (2.2 ± 0.4 vs 2.7 ± 0.3 ml/mmHg, *P* < 0.01).

**Table 1:** Subjects' anthropometric, systemic hemodynamics and left ventricular parameters.

	**Sedentary (*n* = 10)**	**Endurance trained (*n* = 10)**
**ANTRHOPETRIC PARAMETES**		
Age, years	23 ± 2	22 ± 2
Height, cm	174 ± 4	174 ± 8
Body mass, kg	65 ± 7	64 ± 8
Body mass index, kg/m^2^	22 ± 2	21 ± 1
Body surface area, m^2^	1.8 ± 0.1	1.8 ± 0.2
**SYSTEMIC HEMODYNAMICS**		
Heart rate, beats/min	56 ± 6	48 ± 6^**^
Mean arterial pressure, mmHg	82 ± 6	81 ± 5
Brachial systolic BP, mmHg	117 ± 8	116 ± 6
Brachial diastolic BP, mmHg	65 ± 6	63 ± 6
Brachial pulse pressure, mmHg	52 ± 6	53 ± 5
Stroke volume, ml	69 ± 6	84 ± 8^**^
Cardiac output, l/min	3.7 ± 0.3	3.8 ± 0.3
**LEFT VENTRICULAR CHARACTERISTICS**		
End-diastolic diameter, mm	46 ± 2	50 ± 2^**^
End-systolic diameter, mm	30 ± 2	33 ± 3^**^
Fractional shortening, %	35 ± 2	35 ± 3
Ejection fraction, %	64 ± 2	63 ± 5
Mass, g	130 ± 23	189 ± 25^*^
Mass index, g/m^2^	74 ± 11	110 ± 16^**^
Posterior wall thickness, cm	8.8 ± 0.1	9.9 ± 0.1^*^
Interventricular septum thickness, cm	8.1 ± 0.9	1.0 ± 0.1^*^

Values are means ± SD. BP, blood pressure;

* *P < 0.05*,

** *P < 0.01 vs. Sedentary*.

**Table 2:** Subjects' cerebrovascular hemodynamics.

	**Sedentary**	**Endurance trained**
Mean MCA velocity, cm/s	64 ± 7	60 ± 8
Systolic MCA velocity, cm/s	99 ± 10	86 ± 11^*^
Diastolic MCA velocity, cm/s	46 ± 6	42 ± 7
Pulsatile MCA velocity, cm/s	53 ± 6	44 ± 6^*^
Resistive index, mmHg/cm/s	1.27 ± 0.25	1.38 ± 0.20
P_ET_CO_2_, mmHg	40 ± 2	40 ± 3

*Value are means ± SD. MCA, middle cerebral artery; P_ET_CO_2_, partial pressure of end-tidal carbon dioxide*.

* *P < 0.05 vs. Sedentary*.

### LBNP testing

No subject showed the signs of impending presyncope during the LBNP testing. Before SV convert from value recorded via modelflow to value recorded via echocardiogram, we performed two-way ANOVA to verify the presence of significant response difference in SV to LBNP between groups. There was a significant difference between groups (*F* = 6.5, *P* < 0.01) and SV after the LBNP release in ET was significantly larger compared with SED (*P* < 0.01). Reported SV values in all figure and Table 3 were SV after the adjusted value to echocardiogram (calculated SV).

**Table 3:** Responses of systemic and cerebral hemodynamics during baseline, −30 mmHg LBNP and after LBNP stimulation release.

		**Baseline**	**LBNP**	**Release 0–15 s**	**ANOVA** ***P*****-value**		
		**0 mmHg**	**−30 mmHg**		**LBNP**	**Group**	**LBNP × Group**
Heart rate	SED	56 ± 6	63 ± 8	63 ± 7	<**0.001**	**0.003**	0.604
bpm	ET	48 ± 6	55 ± 8	53 ± 5			
SV	SED	69 ± 6^†^	61 ± 6^*^	69 ± 7^†^	<**0.001**	**0.001**	**0.001**
ml	ET	83 ± 8	68 ± 10^*^	88 ± 10^*^			
Cardiac output	SED	3.9 ± 0.3	3.7 ± 0.6	4.0 ± 0.4	<**0.001**	0.440	0.077
l/min	ET	3.9 ± 0.4	3.6 ± 0.3	4.4 ± 0.4			
Aortic PP	SED	32 ± 4	30 ± 4	33 ± 3	<**0.001**	0.294	0.838
mmHg	ET	31 ± 3	28 ± 4	31 ± 3			
Aortic systolic BP	SED	98 ± 9	98 ± 7	99 ± 8	0.580	0.902	0.770
mmHg	ET	97 ± 7	99 ± 12	97 ± 11			
SV/Aortic PP	SED	2.2 ± 0.4^†^	2.1 ± 0.3	2.2 ± 0.4^†^	<**0.001**	**0.005**	<**0.001**
ml/mmHg	ET	2.7 ± 0.3	2.5 ± 0.4^*^	2.9 ± 0.5^*^			
TVC	SED	0.021 ± 0.002	0.023 ± 0.004	0.020 ± 0.004	<**0.001**	0.572	0.239
l/min/mmHg	ET	0.020 ± 0.003	0.023 ± 0.003	0.018 ± 0.004			
Mean MCA	SED	64 ± 7	61 ± 13	61 ± 12	0.104	0.218	0.987
cm/sec	ET	60 ± 8	56 ± 6	56 ± 7			
Pulsatile MCAv	SED	84 ± 12	79 ± 15	87 ± 15	**0.001**	**0.032**	0.669
%	ET	75 ± 10	66 ± 11	78 ± 9			
Systolic MCAv	SED	155 ± 10	152 ± 12	157 ± 13	**0.011**	**0.025**	0.628
%	ET	145 ± 8	142 ± 8	151 ± 8			
Diastolic MCAv	SED	72 ± 4	73 ± 4^†^	70 ± 4	<**0.001**	0.202	**0.006**
%	ET	71 ± 3	76 ± 3^*^	73 ± 2			
Resistive index	SED	1.27 ± 0.25	1.40 ± 0.30	1.37 ± 0.30	**0.026**	0.518	0.968
mmHg/cm/sec	ET	1.38 ± 0.20	1.47 ± 0.22	1.43 ± 0.26			
P_ET_CO_2_	SED	40 ± 2	37 ± 4	37 ± 4	<**0.001**	0.729	0.814
mmHg	ET	40 ± 3	38 ± 3	38 ± 4			

*Values are mean ± SD. Bold values represent P < 0.05. BP, blood pressure; SV, stroke volume; PP, pulse pressure; MCAv, cerebral blood flow velocity; P_ET_CO_2_, partial pressure of end-tidal carbon dioxide; TVC, total vascular conductance*.

* *P < 0.05 vs. baseline*,

† *P < 0.05 vs. ET*.

Table 3 presents the responses of hemodynamics from proximal aorta to cerebral artery to LBNP stimulation and release. HR and cerebrovascular resistance gradually increase associated with increased LBNP pressure in both the groups. Whereas, calculated SV, cardiac output, SV/_Ao_PP, mean and pulsatile MCAv, and P_ET_CO_2_, gradually decrease associated with increased LBNP in both group. At LBNP-30 mmHg, calculated SV does not differ between the groups, whereas cardiac output was maintained. Diastolic MCAv% was significantly elevated at LBNP-30 mmHg in endurance-trained athletes.

**Figure 1:**
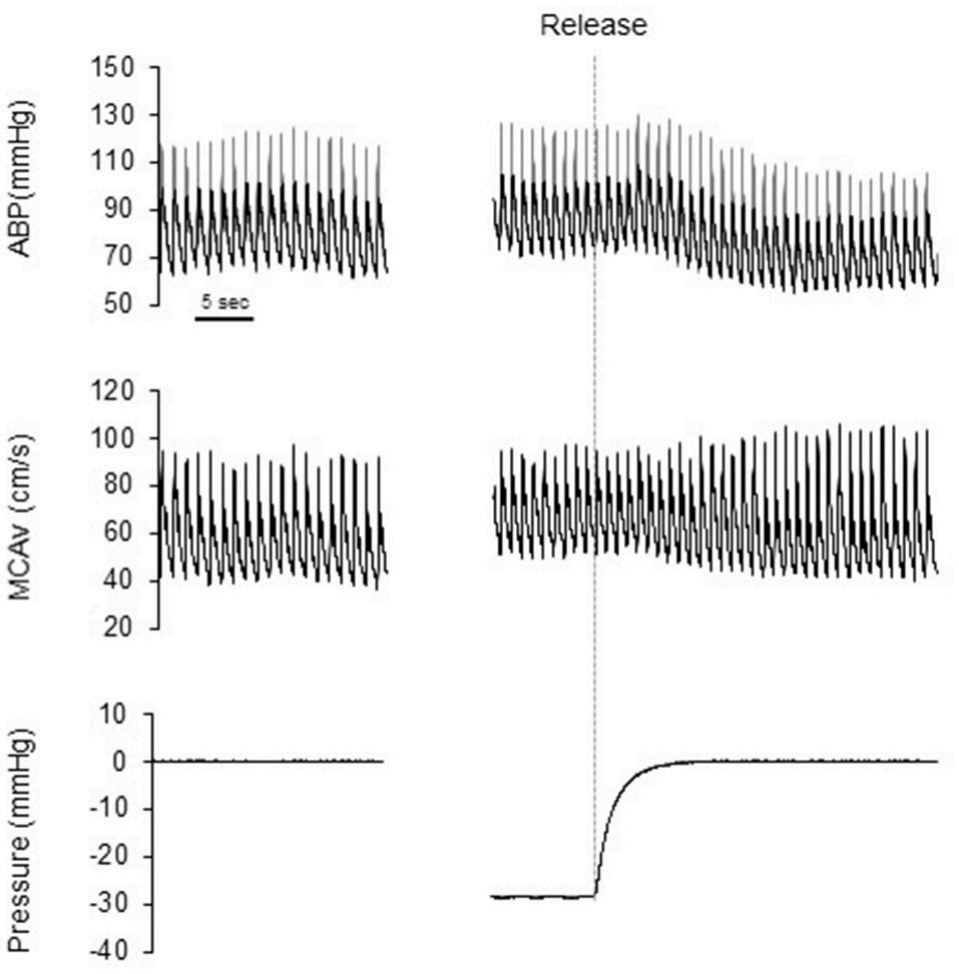
Typical response of radial arterial blood pressure (ABP), transcranial Doppler measured middle cerebral artery blood flow velocity (MCA v) and chamber pressure during baseline (0 mmHg), LBNP-30 mmHg, and after release LBNP.

**Figure 2:**
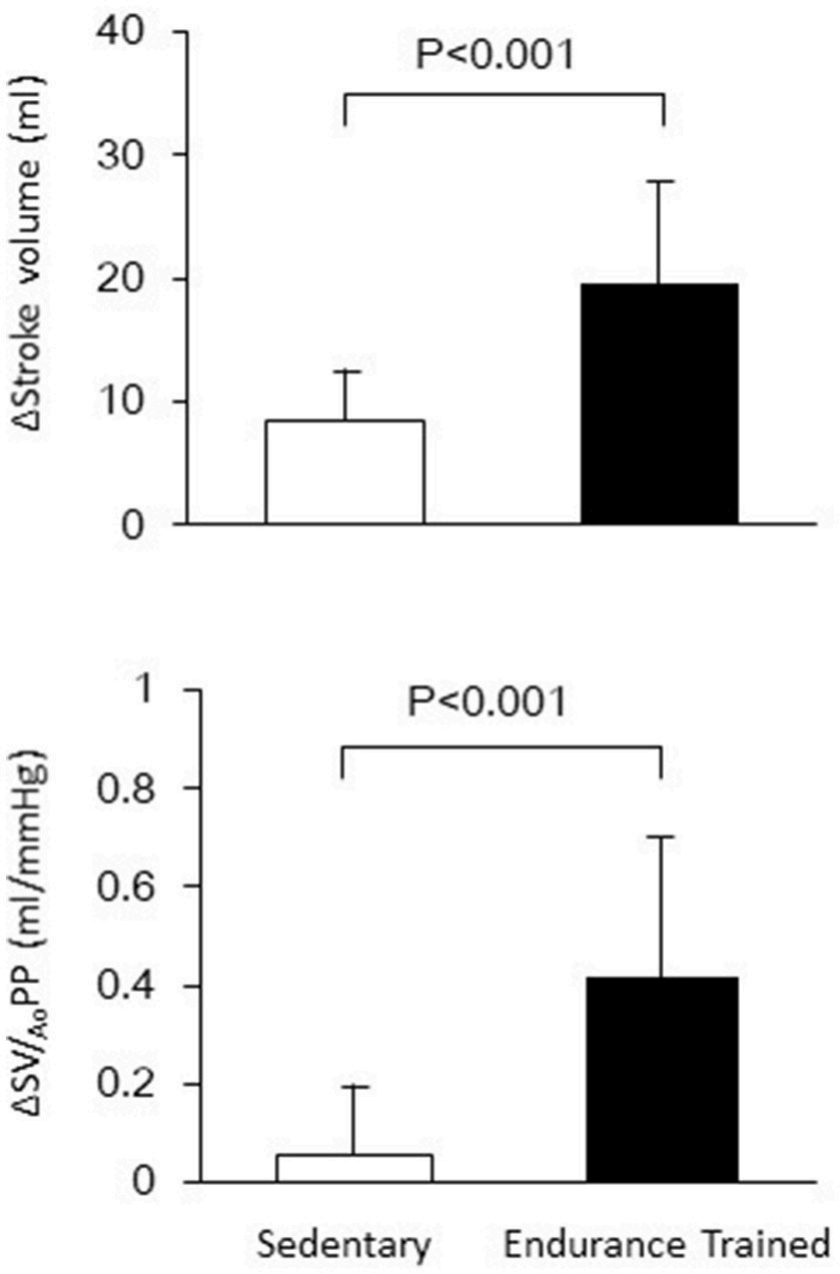
The changes in stroke volume and aortic compliance (SV/_Ao_PP) after the release of LBNP up to 15 s.

**Figure 3:**
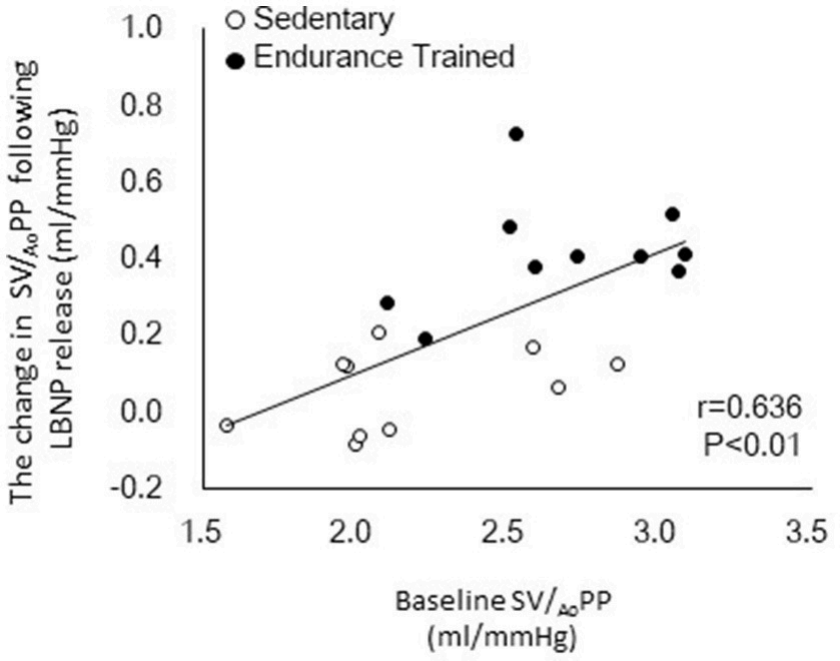
Relationship between the baseline aortic compliance (SV/_Ao_PP) and the change in SV/_Ao_PP following lower body negative pressure (LBNP) release.

**Figure 4:**
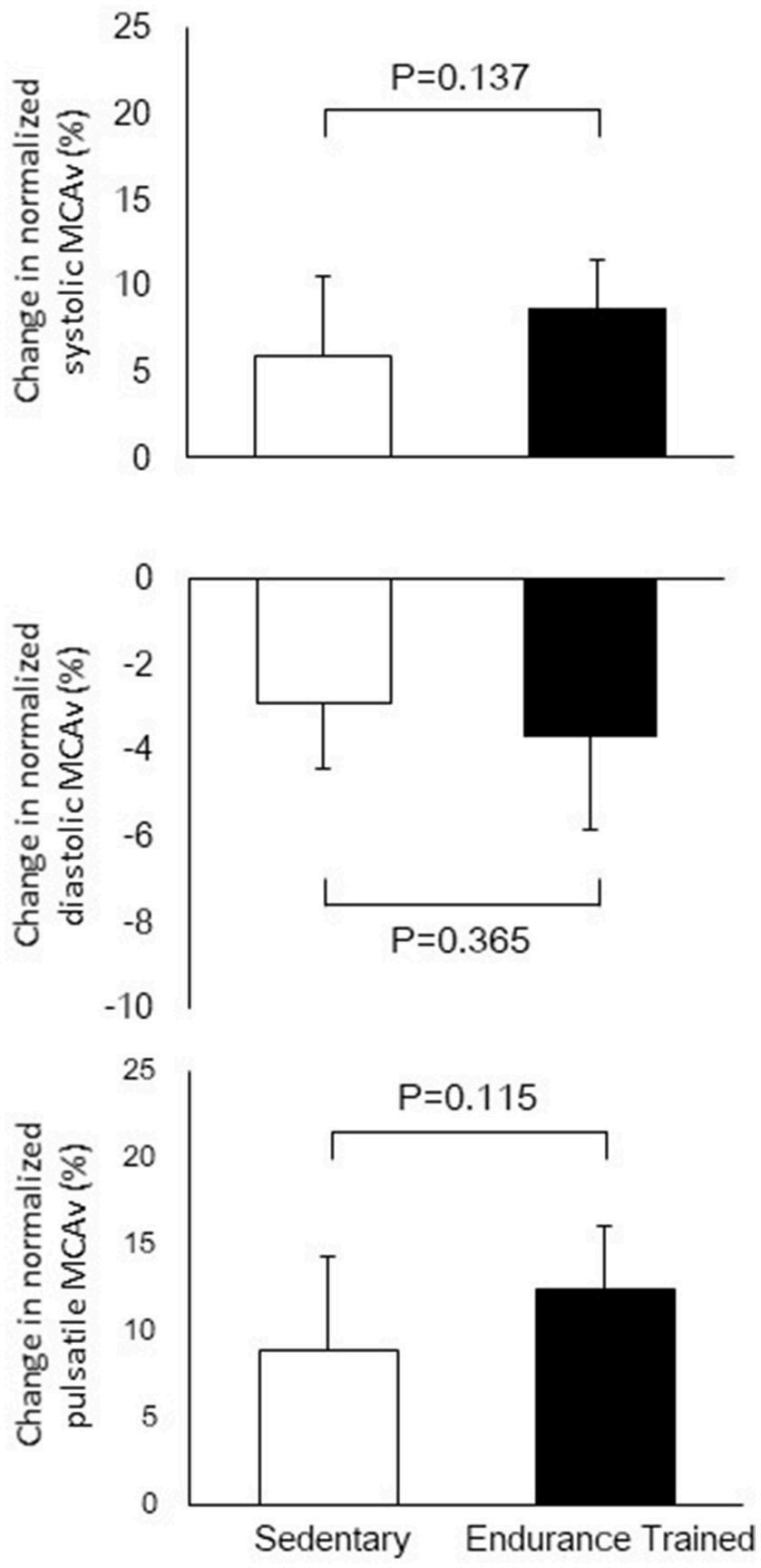
The changes in normalized systolic, diastolic, and pulsatile middle cerebral arterial velocity (MCAv) after the release of LBNP up to 15 s.

Figure 1 shows a typical response of radial arterial blood pressure, MCAv via TCD, and LBNP testing at baseline, at LBNP-30 mmHg and at release up to 15 s. Figure 2 shows the change in calculated SV and SV/_Ao_PP right after the LBNP release. Following the LBNP release, calculated SV was significantly increased in SED by 14 ± 7% and greater in ET by 30 ± 15%, and the change in calculated SV/_Ao_PP was also significantly increased. Importantly, the increase in SV/_Ao_PP following the LBNP release was greater in ET than in SED (*P* < 0.001, Figure 2), and such response significantly correlated with the baseline SV/_Ao_PP (*r* = 0.636, *P* < 0.01, Figure 3). Figure 4 shows the change in normalized systolic, diastolic, and pulsatile MCAv in each group. Although calculated SV significantly increased in ET, no significant differences were observed in normalized MCAv parameters.

## Discussion

The primary findings from the present study are as follows. First, systolic and pulsatile CBF velocity at rest were significantly lower in ET with greater aortic compliance compared with SED. Secondly, SV and pulsatility index of MCA blood flow velocity significantly increased during the release of LBNP in both groups. The increase in SV was significantly greater in ET compared with SED, however, we did not observe a significant group-difference in responses of pulsatile CBF. These results might be attributed to the concomitant increase in aortic compliance assessed by SV/_Ao_PP. Importantly, the increase in SV/_Ao_PP following the LBNP release was greater in ET than in SED and significantly correlated with the baseline SV/_Ao_PP. These results suggest that the aortic compliance in the endurance athletes is able to accommodate the additional SV and buffer the potential increase in pulsatility at end-organs such as the brain.

In this study, SV was significantly increased after the LBNP release (via an acute increase in central blood volume) in both groups. This finding is consistent with previous observation (Zhang et al., 1998; Ogoh et al., 2015). Interestingly, the change in SV was greater in the ET compared with the SED group. This phenomenon might be explained by LV characteristics in the endurance-trained athletes such as exercise-induced cardiac remodeling and myocardial compliance. Increase in the left and right ventricular end-diastolic volume commonly observed in endurance-trained populations (Pluim et al., 2000; Serrador et al., 2000). Additionally, this cardiac remodeling also accommodated with the elimination of LV compliance which reflect to expand LV ventricular with less pressure (Levine et al., 1991; Bhella et al., 2014). According to the Frank-Starling law of the heart, SV increases in response to an increase in the end-diastolic volume which synchronized with the venous return (Guyton and Hall, 2011). Taken together, the LBNP release evoked substantial increased venous return in both groups; however, greater LV capacity with expandable myocardium in ET induced the greater change in SV compared with SED.

The acute increase in blood pulsatile fluctuation at the brain may cause of cerebrovascular damage. Also, physiologically, an acute increase in SV would be a cause of elevation in systolic and diastolic blood pressure and mean arterial pressure, which is mechanical stress at the peripheral organs. Similarly with a change in SV, following the release of LBNP up to 15 s, pulsatility index of MCA velocity significantly increased in both groups. However, there was no significant group-difference in responses of pulsatile CBF despite the greater increase in SV among ET compared with SED. In addition, after the LBNP release, SV/_Ao_PP significantly elevated in ET but not in SED, and the increase in SV/_Ao_PP following the LBNP release was significantly correlated with the baseline SV/_Ao_PP. Taken together, in ET individuals with greater aortic compliance, postural change-related rapid augmentation of cardiac ejection does not cause substantial augmentation of cerebral pulsatile fluctuation.

Cerebral autoregulation may partially take a role of explaining this pulsatile attenuation in cerebral arteries. Cerebral autoregulation reflects the constriction or dilation of cerebrovascular within normal arterial pressure. Dynamic cerebrovascular autoregulation refers to the latency from the start of stimulus to the onset of cerebrovascular conuterregulation. Lind-Holst et al. have reported that endurance-trained individuals appear to weaken dynamic cerebral autoregulation, and this adaptation may increase the risk of symptomatic cerebral hypoperfusion during marked and rapid reductions in blood pressure (Lind-Holst et al., 2011). This hyporeactivity of cerebrovascular may affect to the substantial fluctuation of CBF. Whereas, Ichikawa et al. have reported the no associated with an attenuation in dynamic cerebral autoregulation in endurance training (Ichikawa et al., 2013). According to these inconsistency findings, cerebrovascular autoregulation may have minimal effect on the blood flow fluctuation at CBF.

There is increasing recognition that not only the adequate cerebral perfusion (evaluating by MCAv) but also the attenuated pulsatile fluctuation at cerebrovascular hemodynamics is an important determinant of cerebrovascular health. The change in pulsatile fluctuation may partially accommodate with compliant aorta. The greater amount of evidence has shown the age-related cerebral hypoperfusion and cerebrovascular disease (Davis et al., 1983; Postiglione et al., 1993). Recently, in addition, pulsatile CBF fluctuation was induced by increased in systolic and decrease in diastolic blood flow velocity (Tarumi et al., 2014). Whereas, Zhu et al (Zhu et al., 2013) demonstrated in the cross-sectional study that the lower in systolic and diastolic MCA velocity with lower aortic stiffness were observed in the Masters' athletes compared with age-matched sedentary peers. Our finding provides the evidence of the findings in these previous studies, and the mechanism of endurance exercise preventing pulsatile CBF stress could be explained partly by the greater Windkessl function among endurance trained athletes.

In the present, there are technical limitations that warrant mention. First, TCD measures blood flow velocity in the MCA rather than CBF. Blood flow velocity reflects blood flow only if the diameter of the blood vessel has measured. Early evidence suggested that the diameter of MCA appears to slightly change with LBNP −40 mmHg for 5 min (Serrador et al., 2000). The MCA diameter change would be no greater than in the study (Serrador et al., 2000), and this diameter change would not significantly impact the findings in this research. Secondary, cerebrovascular resistance index was calculated using the mean arterial pressure calibrated by radial signal, not local blood pressure. Most of the invasive human research has this issue. Thirdly, aerobic capacity has not been measured in this study. We focused more cardiac adaptation rather than aerobic capacity; thus, athletic history and recent endurance training status were recorded from each participant to clarify the cardiac adaptation occurred due to prolonged exercise. Fourth, a previous research has reported that there is different response in cardiac output on release of LBNP (Ogoh et al., 2015). Accodring to this, it appears likely that there is a different response in cardiac output between two groups; however, we did not detect the change due to small sample size. Lastly, we studied only apparent small number of healthy young men. To gain generalizability of these findings and a better understanding of the pathophysiology, further studies are needed on the larger number of subjects and other populations such as women, middle-aged and elderly individuals, and patients with impaired Windkessel function or cerebrovascular disease.

In conclusion, we found that systolic and pulsatile CBFV at rest were significantly lower in ET with superior proximal aortic compliance compared with SED group. SV and pulsatility index of MCA blood flow velocity significantly increased after the release of LBNP in both groups. The increase in SV was significantly greater in ET than in SED, whereas there was no significant group-difference in responses of pulsatile CBF. These results might be attributed to the concomitant with the increase in aortic compliance assessed by SV/_Ao_PP. Additionally, SV/_Ao_PP significantly elevated after the LBNP release in ET but not in SED, and the increase in SV/_Ao_PP following a LBNP release was significantly correlated with the baseline SV/_Ao_PP. These results suggest that the aortic compliance in the endurance athletes is able to accommodate the additional SV and buffer the potential increase in pulsatility at end-organs such as the brain.

## Author contributions

TT, TI, SO, and JS conception and design of research; TT, TI, and JS performed experiments; TT, TI, and JS analyzed data; TT and JS interpreted results of experiments; TT and JS prepared figures; TT, SO, SM, and JS drafted manuscripts; TT, TI, SO, SM, and JS approved final version of manuscripts.

### Conflict of interest statement

The authors declare that the research was conducted in the absence of any commercial or financial relationships that could be construed as a potential conflict of interest.
